# The potential to prevent unnecessary emergency department visits by timely diagnosis of migraine–A prospective observational study

**DOI:** 10.1371/journal.pone.0312106

**Published:** 2024-10-18

**Authors:** Hristina Drangova, Nicole Kofmel, Mattia Branca, David Gloor, Beat Lehmann, Aristomenis Exadaktylos, Simon Jung, Urs Fischer, Christoph J. Schankin

**Affiliations:** 1 Department of Neurology, Inselspital, Bern University Hospital, University of Bern, Bern, Switzerland; 2 Department of Emergency Medicine, Inselspital, Bern University Hospital, University of Bern, Bern, Switzerland; 3 CTU Bern, University of Bern, Bern, Switzerland; 4 Department of Neurology and Stroke Centre, University Hospital Basel, University of Basel, Basel, Switzerland; University of the Witwatersrand Johannesburg, SOUTH AFRICA

## Abstract

**Aim:**

Successful acute migraine treatment potentially prevents emergency room (ER) consultations but requires that the diagnosis of migraine was given earlier. The aim of this study is to quantify the problem of missed migraine diagnosis prior to ER visits.

**Methods:**

Inclusion criterion for this single-center prospective study was the presentation at the ER for acute headache. Patients with acute migraine attacks were assessed for previous migraine attacks, and whether they were given a diagnosis of migraine in the past.

**Results:**

Of 137 patients with migraine diagnosis at discharge, 108 (79%) had previous headache attacks fulfilling the criteria for migraine according to The International Classification of Headache Disorders 3rd edition (ICHD-3). Of those, 54 (50%) received the diagnosis for the first time.

**Conclusion:**

Half of the migraine patients (50%) presenting in the ER for headache could have been diagnosed earlier. This highlights the need for better detection and treatment of migraine by pre-hospital healthcare providers, as earlier diagnosis and specific acute treatment could have prevented the ER visit.

## Introduction

As the second most disabling condition worldwide after stroke, and the top ranking cause of disability in Western Europe, migraine imposes a substantial global socioeconomic burden [1]. Moreover, headache is a common chief complaint for presentation at an emergency room (ER) and accounts for approximately 1–4% of all visits and for 8–14% of all consultations at a neurological ER [2–5] worldwide. The majority of these patients, about 75%, suffer from a primary headache attack, mainly migraine, while 25% have secondary headaches [5,6]. Some patients presenting with migraine might have already had multiple migraine attacks beforehand, and can be grouped into two categories: patients with a previous diagnosis of migraine and presumed but not guaranteed access to migraine specific treatment (i.e. with triptans), and patients who have not been diagnosed with migraine, i.e., patients without access to treatment. In the latter group, triptans might have prevented the need to utilize an ER for migraine treatment.

As the prevalence of migraine without an aura is substantial in Switzerland, at around 36% [7], we aimed in this study to quantify the problem of missed diagnosis of migraine prior to ER visits. To do this, we identified patients who presented to the ER with a migraine attack and had already had previous similar attacks, fulfilling the diagnostic criteria for migraine according to The International Classification of Headache Disorders 3rd edition (ICHD-3) [8], without having been diagnosed yet. Worldwide and in Europe [9–12], migraine is known to be underdiagnosed and undertreated. However, to our knowledge, a study examining undiagnosed migraine specifically in Switzerland has not yet been conducted.

## Materials and methods

This single-center prospective observational study was conducted at the University Hospital of Bern, Switzerland from 03/2019 to 11/2021. The study was conducted in accordance with the declaration of Helsinki and approved by the lead cantonal ethics committee (Study Title “Headache in the Emergency Department” (HeED), Ref. number: 2018–01485). All patients either have provided written general consent, or at the very least, have not explicitly declined consent as documented in the written general consent procedure in our institution. Inclusion criteria were age older than 18 years and presentation at the ER with the chief complaint of headache. Primary endpoint was the proportion of patients who had not been given the diagnosis of migraine despite having had fulfilled the diagnostic criteria for migraine in the past.

For this, every patient was interviewed by the ER physician guided by a semi-structured questionnaire that was available to all emergency physicians at our interdisciplinary emergency department, even for routine treatment and not only for the purpose of the study. This questionnaire incorporated the ICHD-3 [8] criteria for migraine with and without aura. It also included a comprehensive list of “red flags” (ex. fever, meningeal irritation, impaired consciousness, mental confusion, epileptic seizure etc.) that may indicate an underlying secondary cause of headache. Depending on the presence of “red flags”, patients were further investigated and most had a minimum of diagnostics (i.e., standard laboratory looking for infectious, inflammatory or metabolic causes for headache, such as CRP, thyroid function, electrolytes, liver and kidney function, and MRI). The diagnosis of every patient was confirmed studying the medical records.

As a second step, the subgroup of patients presenting with the diagnosis of an acute migraine attack was categorized into those who did versus did not have previous headache episodes fulfilling the diagnostic criteria for migraine (ICHD-3) [8]. Further, we assessed if the patient had been given the diagnosis of migraine previously.

Data were collected and analyzed in REDCap (Developer Vanderbilt University, Release 08/2014) using descriptive statistics. As a first step, we distinguished between patients with previous migraine attacks and those without. Patients with previous migraine attacks were dichotomized into two groups, those who had been given the diagnosis of migraine previously and those who have not.

The preliminary results of the study were presented at the International Headache Conference 2021 [13] and at the European Academy of Neurology meeting 2021 [14].

## Results

**Fig 1:**
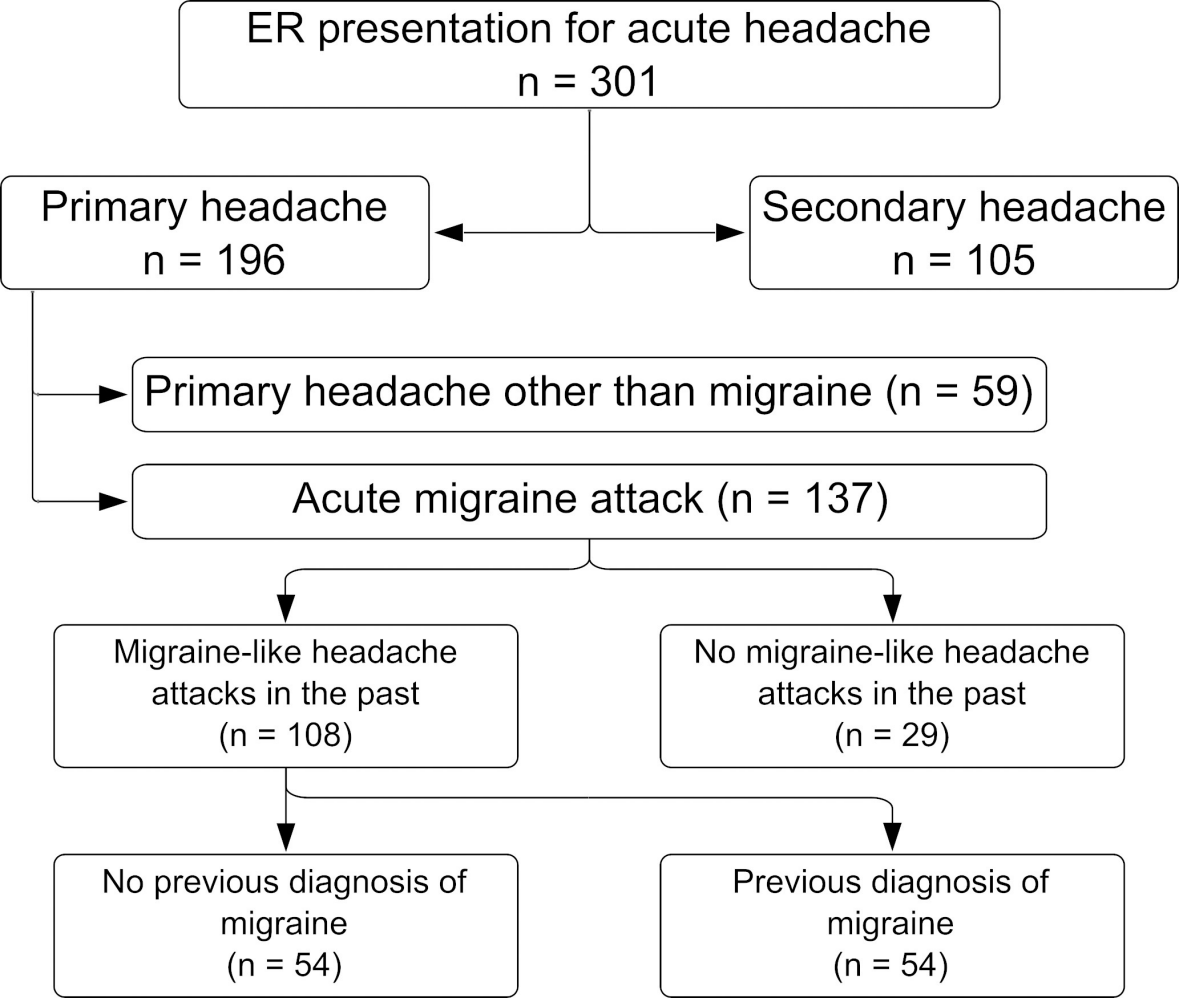
Analysis path of study population.

A total of 301 patients with acute headache as a main symptom were recruited. Among those, 105 (35%) were diagnosed with secondary headache and the remaining 196 (65%) had primary headache (Fig 1). Out of those 196 patients, 137 (70%) were given the diagnosis of acute migraine attacks. Of all migraine patients, 29 (21%) had a first manifestation of migraine, meaning they had no migraine-like headaches in the past. One hundred eight (74% female; mean ±SD age 40.6 ± 15.9) had already had multiple headache episodes in the past fulfilling the ICHD-3 criteria for migraine [8]. Only half of these (n = 54, 50%) have been diagnosed previously with migraine.

## Discussion

The main finding of this study is that most patients who present in an ER for acute migraine attacks have already had previous migraine attacks in the past. However, only about half of these patients have also been given the diagnosis of migraine. In other words, these are undiagnosed migraine patients, who need an ER consultation to be given the correct diagnosis for the first time. This strongly supports that migraine is considerably underdiagnosed in prehospital care [15,16] as also reflected in the utilization of triptans in neighbor countries with similar health care systems [17]. Hypothetically, timely diagnosis with prescription of a specific acute treatment (i.e., triptans) could have prevented the ER visit.

This study furthermore clearly depicts the need of a better recognition, education and awareness regarding migraine in prehospital care. This involves the entire chain of patient care ranging from pharmacists to primary care physicians, neurologists, headache specialists as well as medical laypersons and self-help groups. As migraine is a worldwide burden [1], a timely diagnosis could not only substantially decrease the financial burden for the affected individuals, but also those of employers, healthcare providers and health care insurance companies.

One limitation of this study is that patients had to recall previous headaches in the ER setting raising the possibility of a recall bias. However, we believe that such bias would rather increase the false negative rate, i.e. patients not recalling migraine-like headaches in the past despite having had such headaches suggesting that the number of undiagnosed patients might be even higher. Another limitation is that we have not specifically assessed previous triptan prescription in patients without diagnosis of migraine. We believe that such error might be rather small given that triptans may not be given to patients who do not have a diagnosis of migraine and are underused in the general population of Austria [17]. There is no data on this for Switzerland, but the situation might be similar given the similarity of health care systems. Although we have put considerable efforts in recruiting patients for this study, a substantial proportion of patients with headache at the ER might have been missed due to the demanding atmosphere at the ER. Another limitation to be reflected is the participation of a single ER, and we encourage replicating our results in other countries and health care systems.

## Conclusions

About half of the migraine patients presenting in the ER for headache could have been given the diagnosis earlier. This study clearly demonstrates the need for a better recognition of migraine by pre-hospital healthcare providers. The timely diagnose of migraine and the prescribing of a specific acute treatment (triptans) have the potential to prevent unnecessary ER visits and thus substantially reduce healthcare costs.
